# Prediction of serum anti-HSP27 antibody titers changes using a light gradient boosting machine (LightGBM) technique

**DOI:** 10.1038/s41598-023-39724-z

**Published:** 2023-08-07

**Authors:** Nasrin Talkhi, Mehdi Jabbari Nooghabi, Habibollah Esmaily, Saba Maleki, Mojtaba Hajipoor, Gordon. A. Ferns, Majid Ghayour-Mobarhan

**Affiliations:** 1 Department of Biostatistics, School of Health, Mashhad University of Medical Sciences, Mashhad, Iran; 2 International UNESCO Center for Health-Related Basic Sciences and Human Nutrition, Mashhad University of Medical Sciences, Mashhad, Iran; 3 Department of Statistics, Ferdowsi University of Mashhad, Mashhad, Iran; 4 Department of Mathematical Sciences, University of Copenhagen, 2100 Copenhagen, Denmark; 5 Social Determinants of Health Research Center, Mashhad University of Medical Sciences, Mashhad, Iran; 6 Department of Nutrition Sciences, Varastegan Institute for Medical Sciences, Mashhad, Iran; 7 Division of Medical Education, Brighton & Sussex Medical School, Falmer, Brighton, BN1 9PH Sussex UK; 8 Metabolic Syndrome Research Center, School of Medicine, Mashhad University of Medical Sciences, Mashhad, Iran

**Keywords:** Biochemistry, Cell biology, Chemical biology, Developmental biology, Biomarkers

## Abstract

Previous studies have proposed that heat shock proteins 27 (HSP27) and its anti-HSP27 antibody titers may play a crucial role in several diseases including cardiovascular disease. However, available studies has been used simple analytical methods. This study aimed to determine the factors that associate serum anti-HSP27 antibody titers using ensemble machine learning methods and to demonstrate the magnitude and direction of the predictors using PFI and SHAP methods. The study employed Python 3 to apply various machine learning models, including LightGBM, CatBoost, XGBoost, AdaBoost, SVR, MLP, and MLR. The best models were selected using model evaluation metrics during the K-Fold cross-validation strategy. The LightGBM model (with RMSE: 0.1900 ± 0.0124; MAE: 0.1471 ± 0.0044; MAPE: 0.8027 ± 0.064 as the mean ± sd) and the SHAP method revealed that several factors, including pro-oxidant-antioxidant balance (PAB), physical activity level (PAL), platelet distribution width, mid-upper arm circumference, systolic blood pressure, age, red cell distribution width, waist-to-hip ratio, neutrophils to lymphocytes ratio, platelet count, serum glucose, serum cholesterol, red blood cells were associated with anti-HSP27, respectively. The study found that PAB and PAL were strongly associated with serum anti-HSP27 antibody titers, indicating a direct and indirect relationship, respectively. These findings can help improve our understanding of the factors that determine anti-HSP27 antibody titers and their potential role in disease development.

## Introduction

Intracellular protective proteins known as heat shock proteins (HSPs) are expressed in response to stressful situations within cells and enable the cells to overcome these conditions^1^. These stressful conditions include environmental, physical, and chemical stressors such as high temperature, viral infections, oxidative stress, ischemia, toxins, and reactive oxygen species^1^.

Heat shock proteins play a molecular chaperone role in the body and are found in most cells in the body. These molecular chaperones are used in the refolding of damaged cell proteins and prevent the accumulation of fat in certain pathways^2^. HSP27 is a small HSPs with a molecular mass of 27 kDa^3^. A high serum concentration of several HSPs has been reported in individuals with cardiovascular disease (CVD)^1^. The overexpression of HSPs, including HSP27 in the body is not good and it causes the immune system to recognize it as an auto-antigen and thus release an antibody called anti-HSP27 against it^4^. Therefore, an increase in HSP27 and consequently an increase in antibodies produced against it (anti-HSP27) has been expressed as an inflammatory marker in the body^1^. Identify the factors associated with serum anti-HSP27 antibody titers is important.

Recent studies have investigated this topic. For instance, in^5^ the authors investigated the association between the serum anti-HSP27 antibody level and the presence of the metabolic syndrome. In^6^ the results showed a significant correlation between vitamin D and anti-HSP27 antibody titers. Moreover, they found that serum pro-oxidant-antioxidant balance was positively associated with serum anti-HSP27 antibody titers. The relationship between the serum anti-HSP27 antibody titers and diabetes was not significant in^7^. A significant direct relationship has been observed between some factors such as hypertension^8^, obesity, body mass index, age, height, serum LDL-C, serum triglycerides, and serum total cholesterol^1^, hypertriglyceridemia^9^ with serum anti-HSP27 antibody titers.

The real-world data in many fields of health, education, and the social sciences yield values of skewness and kurtosis that clearly deviate from the normal distribution. Bono et al. mentioned some of non-normal variables that were listed by Arnau et al. such as, reaction times or response latency in cognitive studies, survival data from clinical trials, clinical assessment indexes in drug abuse research, physical and verbal violence in couples, divorced parents’ satisfaction with co-parenting relationships in family studies, and labor income or health care costs in sociological studies. More recent examples involving non-normal data include neuropsychological data, data about paranoid ideation, fatigue symptoms of breast cancer patients, data on violence or sexual aggression, and numerous studies on the cost of health care, such as costs among patients with depression or anxiety, costs following brief cognitive behavioral treatment for insomnia, and costs of anorexia nervosa^10^. Hence, an useful algorithm must be used to construct the model to take this into account^11^. Machine learning methods can be used for these problems. The process of applying new methods to discover knowledge behind data using a computer is called data mining which uses machine learning techniques. Generally, we face two types of machine learning methods, supervised and unsupervised^12^.

Today, machine learning techniques have become increasingly popular in medical research. There is growing evidence that these methods can be effectively applied to a wide range of real-world problems in the field^13–15^.

Jing et al.^16^ used machine learning classification algorithms to predict and categorized HSPs into six different families, HSP20, HSP40, HSP60, HSP70, HSP90, and HSP100. They applied a support vector machine (SVM) to achieve this purpose. Another published study that has conducted by Meher et al. has the same goal with Jing’s study^17^.

Min et al. proposed a convolutional neural network (CNN) that classifies both non-HSPs and six HSP families simultaneously. Their algorithm was trained on raw protein sequences and also on top of pre-trained protein representations^18^. Moreover, Chen et al. also used the two benchmark datasets of HSPs and their goal was the classification of HSPs and then prediction them using machine learning algorithms^19^.

We did not find any studies that used machine learning techniques for the prediction of serum anti-HSP27 antibody levels using related factors such as demographic factors, chronic diseases, social factors, chemical parameters, etc. In previous studies, simple methods such as general linear models, case–control studies^1,20^, logistic regression, and simple statistical tests such as correlation tests, analysis of variance, Kruskal–Wallis test^1,5–9^ were used.

The strengths of this study can be mentioned as follows: I) This study has been done on a larger scale with a population of 4181, II) Some of the attributes studied in this study have not been studied in previous studies, III) Using the advanced machine learning methods, V) So far, no study has been done in this area using machine learning techniques.

**Figure 1:**
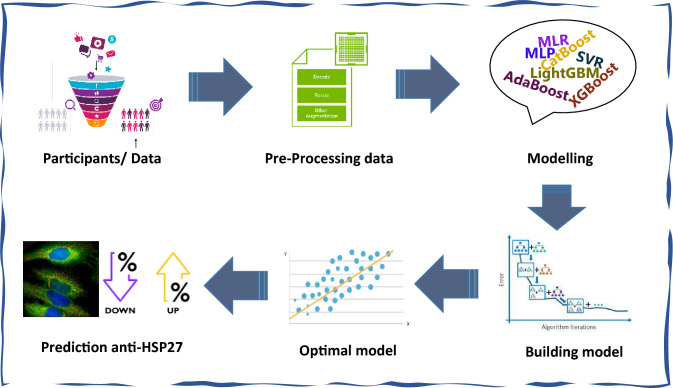
Graphical Abstarct.

This paper is organized as follows. In section “Introduction”, we discussed HSPs, anti-HSP27, and a brief review of the related conducted studies in this area. Section “Material and methods”, i.e. material and methods, we expressed a brief demonstration of machine learning methods and their application. In section “Results”, the results of the research are reported. Section “Discussion” discusses the results obtained and compare them to other related studies. Finally, section “Conclusion” concludes this study. The graphical abstract is shown in Fig. 1.

## Material and methods

### Study design, data collection, and data processing

The present study recruited participants from the Mashhad stroke and heart atherosclerotic disorder (MASHAD) cohort study at baseline^21^. Inclusion criteria required participants to be between the ages of 35 and 65, while exclusion criteria involved being outside this age range or declining to participate in the study. A total of 9,074 individuals who met the inclusion and exclusion criteria were enrolled in the study.

In the pre-processing phase, after removing the missing values, the 4,181 complete data records remained. Moreover, the data were normalized using the normalization formula as follows:

$${X}_{normalized}=\frac{{X}_{i}-{X}_{i,minimum}}{{X}_{i,maximum}-{X}_{i,minimum}}$$

where, ${X}_{i}$ refer to the actual value and ${X}_{normalized}$ contains the normalized values of the X variable. Some used algorithms in this study such as SVM, MLP, and MLR need to be normalized, but the LightGBM does not. To develop the desired set of machine learning models, we used the K-Fold cross-validation (CV) methods on data. The required hyperparameters of models were determined during the fivefold CV method according to Table 3.

All analyses related to the pre-processing and modelling were implemented using Python 3 programming language.

### Predictive techniques

#### Multiple linear regression (MLR)

Linear regression is one of the most common predictive models so it is the basis of regression-based machine learning models as well as, is so popular, simple, and widely used. In fact, this model tries to predict the outcome values using some predictors. In other words, it studies the relationship between predictor variables and outcome^22^.

#### Multilayer perceptron (MLP)

One of the most used and popular methods in machine learning is Multilayer perceptron (MLP) which is almost simple and has clear architecture. MLPs are neural networks that include at least three layers. This model consists of inputs, weights, biases, and an activation function that yields the output. The neurons of a certain layer feed the neurons of the next layer with their outputs. The connection power between neurons is determined by the adaptive coefficient weights, which are multiplied by each input to neurons. After that, a non-linear function i.e. activation function (usually applying sigmoid or hyperbolic tangent function) is used. The training process consists of adjusting the coefficients (weights) of the MLP. With calculating the error function (mean value of the difference between the actual target (T) and the forecasted output) and updating weights based on the learning rate and the error in each epoch and at the end repeating steps until reaching the number of epochs, the training process is completed and the final weights are determined^23^.

#### Support vector machine (SVM)

SVMs are one of the supervised learning methods and they are used for classification and regression problems^24^ and recently have been successfully acted in solving these problems^25^. In classification problems, SVMs create optimal decision boundaries between observations of two or more classes, and in Regression or approximation function problems, SVMs approximate optimal function to data. In both approaches, SVMs are found the optimal solution for solving a quadratic optimization problem. The SVMs for classification are called Support Vector Classification (SVC) and the SVMs for regression are called Support Vector Regression (SVR)^11^. Also, SVMs use various kernel functions to choose optimal non-linear decision boundaries in classification and optimal non-linear functions in regression^11,26,27^. Unlike common statistical methods, SVMs do not need to know the probability distribution of observations and also, unlike Neural networks, SVMs have an optimal and global solution. On the other, in SVMs, the complexity of the calculations does not depend on the number of input variables^11,28^. SVMs based on the structural risk minimization principle try to minimize the upper bound of generalization error and this is the final goal of SVM^11,12,26^.

#### Ensemble methods

One of the branches of machine learning is ensemble methods. Ensemble methods combine some weak learners to build a reliable model. The main goal of ensemble learning is to improve predictability in models. In other words, converting weak learners to strong learners, increase the accuracy of the results significantly. As well, ensemble learning can handle classification and regression problems well and are ideal. The popularity of it is due to reducing the bias and variance to boost the accuracy of models^29^.

Weak base learners in ensemble learning can be homogenous (base weak learners of the same types) or heterogeneous (base weak learners of the different types). Ensemble learning methods are mainly divided into categories of boosting and bagging. Bagging stands for bootstrap aggregating such as random forest (RF). Boosting has various forms such as gradient boosting, adaptive boosting (AdaBoost), categorical boosting (CatBoost), light gradient boosting machine (LightGBM), and extreme gradient boosting (XGBoost) algorithms^29^.

An improved version of the gradient boosting algorithm is called XGBoost and is one of the popular machine learning algorithms. This algorithm works based on the decision tree approach and the gradient boosting decision tree algorithm is the base it exactly. This algorithm has powerful predictive power and its implementation is simple^30^.

**Figure 2:**
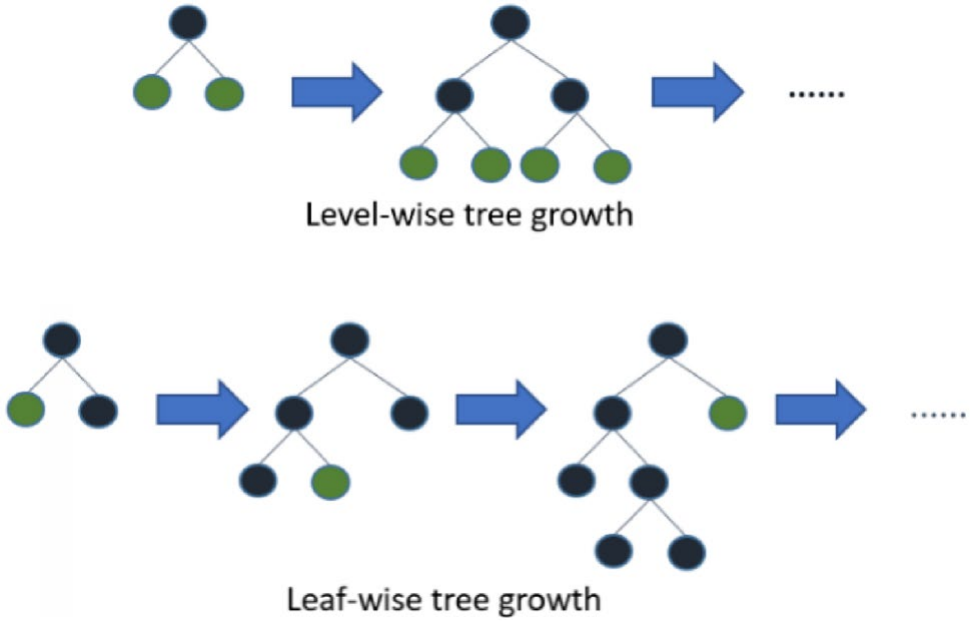
Presentation of level-wise versus leaf-wise growth strategy.

Ke et al. proposed an ensemble model in 2017^31^. This model works based on a decision tree algorithm as a weak learner that was called LightGBM. It uses a novel technique called histogram-based binning and learns more efficiently than other algorithms. Tree-based models such as XGBoost produce the trees by level-wise growth method. While LightGBM applies the leaf-wise growth strategy rather than the level-wise growth method to generate the tree (Fig. 2).

Applying the leaf-wise growth strategy rather than the level-wise growth method can reduce the errors and then leads to high accuracy. In addition, LightGBM can handle high-dimensional problems. The generated decision tree with XGBoost is made using a level-wise growth method^30,32^. CatBoost is a supervised machine learning method based on gradient boosting on decision trees that is a powerful method and appropriate for the classification and regression problem with a dataset consisting of many categorical variables. AdaBoost is another ensemble method. The most common weak learner in adaBoost is the decision tree. It is the first successful algorithm to boost binary classification^32^.

### Model evaluation

For evaluating the performance of models, we employed five performance metrics. The formula of these metrics is expressed as follows:

- Root Mean Square Error
  $$RMSE=\sqrt{\frac{1}{N}\sum_{i=1}^{N}{({y}_{i}-{\widehat{y}}_{i})}^{2}}$$
- Mean Absolute Error
  $$MAE=\frac{1}{N}\sum_{i=1}^{N}|{y}_{i}-{\widehat{y}}_{i}|$$
- Coefficient of Determination
  $${R}^{2}=1-\frac{\sum_{i=1}^{N}{({y}_{i}-{\widehat{y}}_{i})}^{2}}{\sum_{i=1}^{N}{({y}_{i}-{\overline{y} }_{i})}^{2}}$$

### Explanation techniques

#### Explanations using SHAP

With respect to most of the machine learning methods are black boxes, therefore, it leads to difficult interpretation. Hence, we need Explainable Machine Learning methods. SHAP stands for "SHapley Additive exPlanations". Shapley values are a widely used approach based on a game theory to explain the output of machine learning models. It technique provides global interpretability, i.e., SHAP values not only show feature importance but also show whether the feature has a positive or negative impact on predictions. In other words, this method approximates the individual contribution of each feature, for each row of data. It approximates the contribution of that feature by estimating the model output without using it versus all the models that do include it^30,33^.

#### Feature importance using PFI

Fisher et al. proposed the Permutation Feature Importance for the random forest. After that, this extended and can be applied to all machine learning methods. The values of a variable are permuted to assess prediction error increases or decreases via permutation. In this method, the relationship between the desired variable and outcome is broken and then the decrease in the evaluation metrics shows how much the model depends on the feature. In fact, this method shows how important this feature is for a desired machine learning method^30,34^.

### Ethics approval and consent of participant

The study protocol was given approval by the Ethics Committee of Mashhad University of Medical Sciences and written informed consent was obtained from participants. All methods were conducted in accordance with relevant guidelines and regulations. Ethic approval code: IR.MUMS.MEDICAL.REC.1399.558.

## Results

We included 55 attributes from the database. Descriptive statistics and the bivariate analysis for evaluating the initial association between the target variable and all of the independent variables are reported in Tables 1 and 2. We used the Kolmogorov–Smirnov (Lilliefors correction) test to check the normality of the distribution. Due to the non-normal distribution of variables, we used the non-parametric Spearman correlation coefficient test, the Mann–Whitney U test, and the Kruskal–Wallis H test. The mean and standard deviation of the anti-HSP27 variable are 0.246 and 0.177, respectively.

**Table 1:** Descriptive Statistics for quantitative clinical and biochemical characteristics of the study population.

Variable name	Abbreviation	Mean ± SD	*P*-value
Anti-heat shock protein 27 (optical density)	Anti-HSP27	0.257 ± 0.196	–
Triglycerides (mg/dl)	TG	143.637 ± 91.578	0.528
Cholesterol (mg/dl)	Chol	192.570 ± 38.945	− 0.524
Low-density lipoprotein (mg/dl)	LDL	118.070 ± 34.968	0.442
High-density lipoprotein (mg/dl)	HDL	42.784 ± 9.853	0.002**
Pro-oxidant-antioxidant Balance (H2O2%)	PAB	69.275 ± 57.219	< 0.001***
Physical activity level	PAL	1.595 ± 0.284	0.101*
Systolic blood pressure (mm Hg)	SBP	122.637 ± 18.344	0.121*
Diastolic blood pressure (mm Hg)	DBP	79.554 ± 11.198	0.999
Body mass index (kg/${\mathrm{m}}^{2})$	BMI	27.919 ± 4.719	0.022**
High sensitivity C-reactive (mg/dl)	Hs-CRP	1.600 (2.480)#	0.631
Age (year)	Age	48.626 ± 7.875	0.016**
Waist circumferences (cm)	WC	95.876 ± 11.941	0.005**
Hip circumferences (cm)	HC	103.863 ± 9.165	0.141*
Waist-to-hip ratio	WHR	0.923 ± 0.080	0.003**
Mid-Upper Arm Circumference (cm)	MUAC	30.619 ± 3.649	0.036**
Glucose (mg/dl)	Glucose	93.413 ± 39.289	0.033**
White blood cells ($\times {10}^{3}/\mathrm{\mu l}$)	WBC	6.069 ± 1.513	0.625
Red blood cells (${\times 10}^{6}/\mathrm{\mu l}$)	RBC	4.844 ± 0.468	0.759
High hemoglobin count (g/dl)	HGB	13.734 ± 1.476	0.426
Red cell distribution width (%)	RDW	41.577 ± 3.124	0.117*
Platelet distribution width (fl)	PDW	12.752 ± 2.011	0.317
Neutrophils to lymphocytes ratio	NL	1.635 ± 1.248	0.060*
Hematocrit (%)	HCT	41.178 ± 3.788	0.662
Platelet Count ($\times {10}^{3}/\mathrm{\mu l}$)	PLT	231.996 ± 60.575	0.188*
Lymphocytes percent (%)	LYMP	36.118 ± 7.344	0.047**

^#^ Median (IQR or Interquartile Range); ***p-value < 0.001; **p-value < 0.05; *p-value < 0.2.

**Table 2:** Descriptive Statistics of qualitative clinical and biochemical characteristics of the study population.

Variable Name	Abbreviation	Levels	N (%)	*P*-value
Diabetes	Diabetes	< 126	3818 (85.1)	0.140*
		> = 126	668 (14.9)	
Obesity	Obesity	< 30	3096 (69.0)	0.035**
		> = 30	1390 (31.0)	
Gender	Gender	Male	1818 (40.5)	0.917
		Female	2668 (59.5)	
Chronic obstructive pulmonary disease	Pulmonary	No	4067 (90.7)	0.130*
		Yes	419 (9.3)	
Autoimmune disease	Autoimmune	No	4385 (97.7)	0.107*
		Yes	101 (2.3)	
Hypertension disease	HTN	HTN^-^	3006 (67.0)	0.092*
		HTN^+^	1480 (33.0)	
Education level	Education	Low	2507 (55.9)	0.014**
		Moderate	1476 (32.9)	
		High	503 (11.2)	
Job status	Job	Student	7 (0.2)	0.006**
		Employment	1626 (36.2)	
		Un-employment	2389 (53.3)	
		Retired	464 (10.3)	
Marital status	Marriage	Single	28 (0.6)	0.440
		Married	4160 (92.7)	
		Divorced	62 (1.4)	
		Widow	236 (5.3)	
Smoking status	Smoking	Non-smoker	3060 (68.2)	0.085*
		Ex-smoker	443 (9.9)	
		Current-smoker	983 (21.9)	

**p-value < 0.05; *p-value < 0.2.

The hyperparameters tuning to achieve the optimal models was performed using fivefold CV and the optimal values of hyperparameters were summarized in Table 3. The obtained evaluation metrics values by MLR, SVM, MLP, LightGBM, XGBoost, CatBoost, and AdaBoost during the training and test phases were shown in Table 4 as mean ± standard deviation.

**Table 3:** The hyperparameters tuning of models.

Parameters	Definition	LightGBM	XGBoost	CatBoost	AdaBoost	SVR	MLP
Boosting_type	Boosting method	gbdt	–	–	–	–	–
Learning_rate	Boosting learning rate	0.01	0.1	0.01	0.001	–	–
Max_depth	Maximum tree depth for base learners	5	3	7	–	–	–
Min_child_samples	Minimum number of data needed in a child	100	–	–	–	–	–
Num_leaves	Maximum tree leaves for base learners	30	–	–	–	–	–
Subsample	Subsample ratio of the training instance	0.1	0.5	0.7	–	–	–
Booster	Type of booster	gbtree	–	–	–	–	–
Gamma	Minimum loss reduction	–	–	–	–	–	–
n_estimator	Number of gradient-boosted trees	–	–	500	100	–	–
Min_child_weight	The minimum sum of instance weight(hessian)	–	5	–	–	–	–
Loss	Loss function	–	–	–	Linear	–	–
Kernel	Kernel type	–	–	–	–	rbf	–
C	Regularization parameter	–	–	–	–	10	–
Gamma	kernel parameter	–	–	–	–	0.08	–
Epsilon	The epsilon-tube	–	–	–	–	0.01	–
Hidden_layer_sizes	Number of neurons in the ith hidden layer	–	–	–	–	–	20
Max_iter	The maximum number of iterations	–	–	–	–	–	20
Learning_rate_init	The initial learning rate	–	–	–	–	–	0.08
Learning_rate	Learning rate	–	–	–	–	–	Invscaling
Activation	Activation function	–	–	–	–	–	relu

**Table 4:** Evaluation of trained models.

Model	Train			Test		
	RMSE	MAE	MAPE	RMSE	MAE	MAPE
LightGBM	0.1914 ± 0.0014	0.1461 ± 0.0013	0.9237 ± 0.0285	0.1900 ± 0.0124	0.1471 ± 0.0044	0.8027 ± 0.064
XGBoost	0.1752 ± 0.0011	0.1322 ± 0.0012	1.3979 ± 0.052	0.1977 ± 0.0065	0.1485 ± 0.0042	1.5565 ± 0.1633
CatBoost	0.1787 ± 0.0013	0.1346 ± 0.0011	1.3992 ± 0.0525	0.1991 ± 0.0095	0.1473 ± 0.0043	1.5212 ± 0.1643
AdaBoost	0.1945 ± 0.0017	0.1461 ± 0.0013	1.5437 ± 0.0476	0.1957 ± 0.0073	0.1504 ± 0.0030	1.5510 ± 0.1684
SVR	0.1828 ± 0.0091	0.1386 ± 0.0045	1.2952 ± 0.0590	0.1954 ± 0.0073	0.1475 ± 0.0048	1.6571 ± 0.1212
MLP	0.1961 ± 0.0017	0.1433 ± 0.0016	0.7828 ± 0.0245	0.1975 ± 0.0075	0.1499 ± 0.0075	0.9246 ± 0.1048
MLR	0.1806 ± 0.0076	0.1409 ± 0.0057	1.4872 ± 0.1653	0.1955 ± 0.0075	0.1479 ± 0.0058	1.6607 ± 0.3333

The mean ± standard deviation was reported.

According to these results, the performance of LightGBM was assessed as RMSE = 0.1914, MAE = 0.1461, and MAPE = 0.9237 on the training and RMSE = 0.1900, MAE = 0.1471, and MAPE = 0.8027 on the test dataset. This model outperformed other models on unseen data (test dataset) significantly.

The XGBoost model (RMSE = 0.1752, MAE = 0.1322, MAPE = 1.3979) performed better than other models, after that, the CatBoost (RMSE = 0.1787, MAE = 0.1346, MAPE = 1.3992) was performed superior to those of AdaBoost (RMSE = 0.1945, MAE = 0.1461, MAPE = 1.5437), SVR (RMSE = 0.1828, MAE = 0.1386, MAPE = 1.2952), MLP (RMSE = 0.1961, MAE = 0.1433, MAPE = 0.7828), and MLR (RMSE = 0.1806, MAE = 0.1409, MAPE = 1.4872) models in the training.

**Figure 3:**
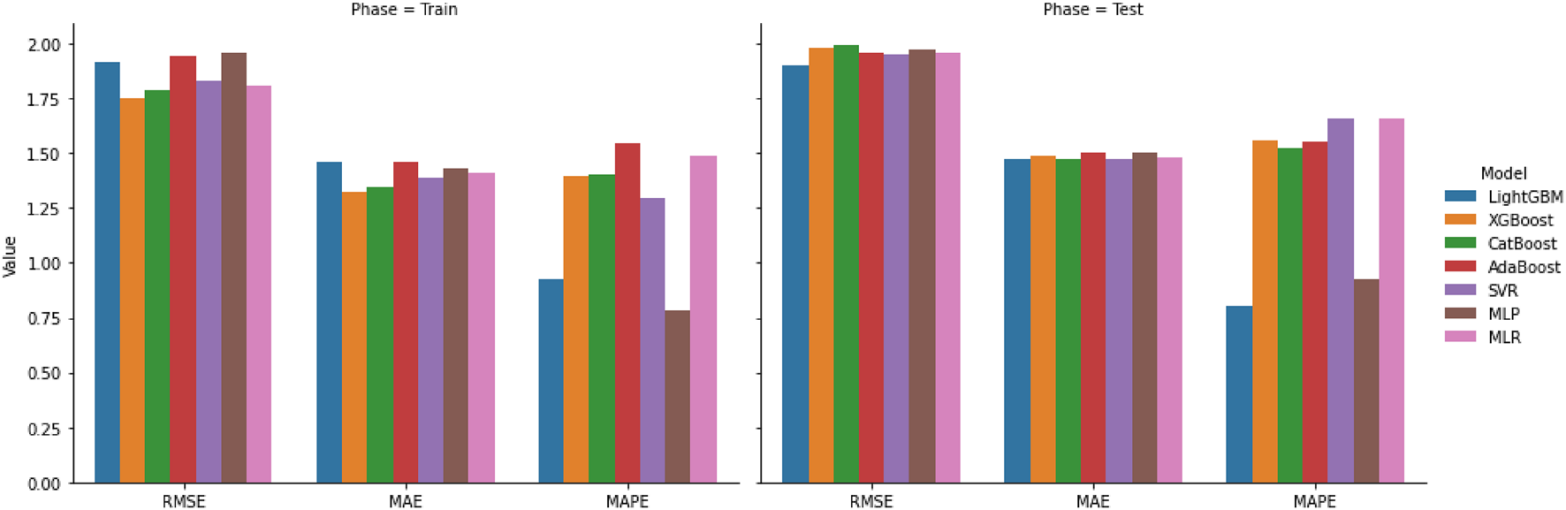
Barplot for model evaluation (RMSE and MAE are multiplied by 10 for better visualization).

In the test phase, the other models such as the XGBoost (RMSE = 0.1977, MAE = 0.1485, MAPE = 1.5565), CatBoost (RMSE = 0.1991, MAE = 0.1473, MAPE = 1.5212), AdaBoost (RMSE = 0.1957, MAE = 0.1504, MAPE = 1.5510), SVR (RMSE = 0.1954, MAE = 0.1475, MAPE = 1.6571), MLP (RMSE = 0.1975, MAE = 0.1499, MAPE = 0.9246), and MLR (RMSE = 0.1955, MAE = 0.1479, MAPE = 1.6607) were evaluated (See the Table 4). These values also are visualized in the bar chart in Fig. 3.

During the model evaluation, the LightGBM was recognized as the most accurate model. Then, we explained the predictions of the most accurate model using two model-agnostic explanation techniques: permutation feature importance (PFI) and shapley additive explanations (SHAP).

**Figure 4:**
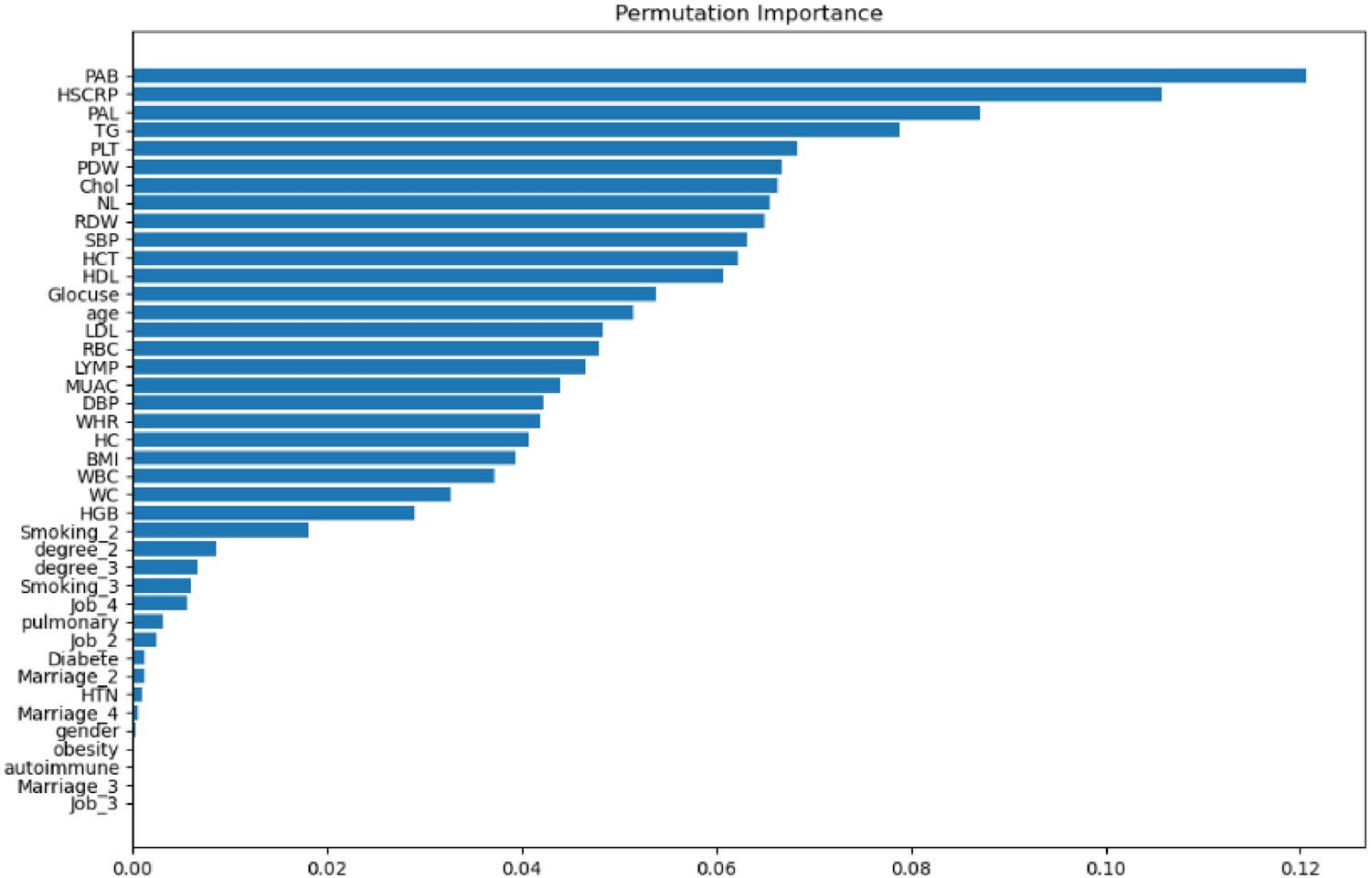
PFI scores of the studied features.

The plotted bar chart in Fig. 3, shows the feature importance (for each feature) in the estimation of antibody titers using the PFI technique. Among all features, the PAB, HS-CRP, PAL, and TG were identified as the four most important features. The importance order of other features are shown in Fig. 4.

**Figure 5:**
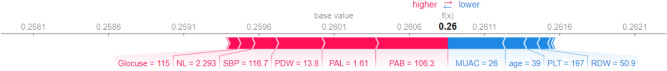
Explanation of the LightGBM model’s output value of 0.26 using SHAP.

The base value in Fig. 5 indicates the mean LightGBM model prediction. Some features were presented in red and some in blue. The red (blue) features move the estimation higher (lower) than the base value. The LightGBM model’s output value is 0.26.

**Figure 6:**
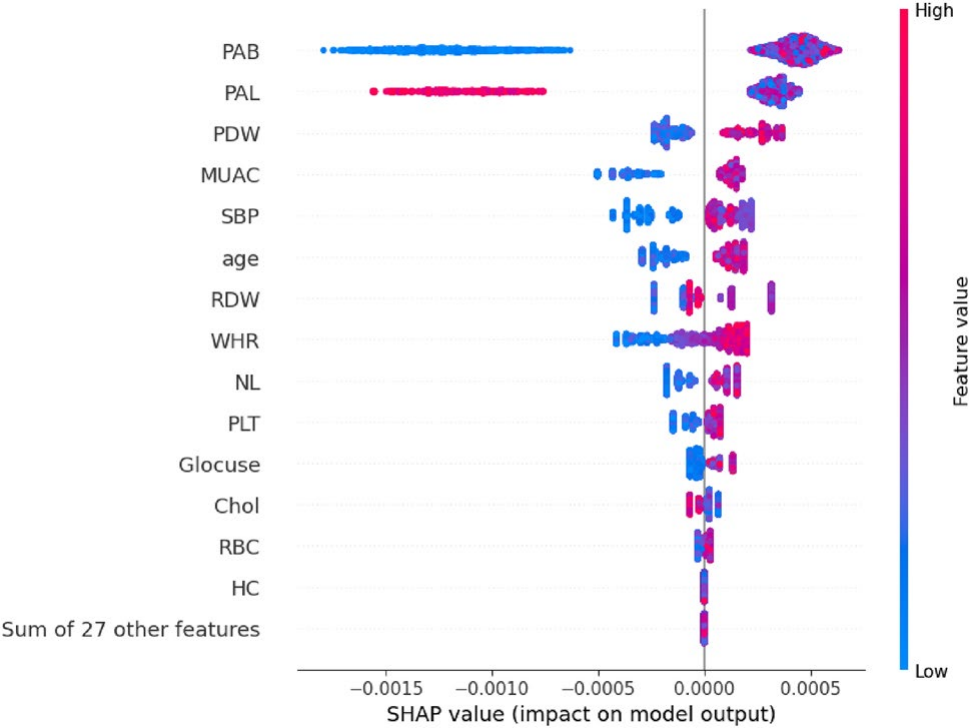
SHAP summary plot of the LightGBM model.

The global explanation was offered via the PFI score. We used the SHAP summary plot (Fig. 6) to explain both local and global explanations and show whether the feature has a positive or negative impact on predictions. Using this plot, we can measure the magnitude and direction. The PAB and PAL were identified as the most effective variables in serum anti-HSP27 antibody titer prediction using PFI and SHAP.

The low values of the PAB variable have a high negative contribution to the prediction, while high values have a high positive contribution. The high values of the PAL variable have a high negative contribution to the prediction, while low values have a high positive contribution.

The PDW, MUAC, SBP, age, RDW, WHR, NL, PLT, Glocuse, and RBC variables have a negative contribution when their values are low, and a positive contribution on high values. While, high values of the Chol have a negative contribution to the prediction and also, low values have a positive contribution. The RBC has an almost modest contribution. HC and the 27 other features have almost no contribution to the prediction.

## Discussion

Data mining and the use of machine learning methods in various scientific fields have made significant progress in their methodologies. The field of data mining research includes powerful processes and tools that lead to the effective analysis and knowledge discovery. Data mining aims are to discover patterns and unknown correlations and predict data trends and behaviors^35^. Ensemble methods are powerful data mining tools^24^.

Elevated serum levels of several heat shock proteins (HSPs), including HSP27, have been observed in individuals with cardiovascular disease (CVD). However, excessive expression of HSP27 can have detrimental effects, leading to increased inflammation in the body. Identifying the associated variables with serum anti-HSP27 antibody levels can serve as a potential biomarker of inflammation in individuals with CVD. Understanding the underlying mechanisms of CVD and identifying such biomarkers can aid in the development of new therapeutic strategies for treating and managing CVD. Overall, this study has important implications for improving our understanding of CVD pathogenesis and advancing the development of effective treatments.

In the present study, the LightGBM model that it is a combination of decision trees as the weak learners was applied. Using a data mining approach, this study represents the first attempt to identify the demographic, clinical, and biochemical characteristics associated with anti-HSP27. The study's strengths include the use of advanced and novel methods, as well as a large sample size.

Our results showed that variables pro-oxidant-antioxidant balance (PAB), physical activity level (PAL), platelet distribution width (PDW), mid-upper arm circumference (MUAC), systolic blood pressure (SBP), age, red cell distribution width (RDW), waist-to-hip ratio (WHR), neutrophils to lymphocytes ratio (NL), platelet count (PLT), glucose, cholesterol, red blood cells (RBC) were associated to anti-HSP27.

The relative importance of variables showed that the PAB was the most important and related variable to serum anti-HSP27 antibody titers with a direct effect on the prediction of serum anti-HSP27 antibody titers. Ghazizadeh et al.^6^ for investigating the relationship between serum anti-HSP27 antibody titers and RDW, PAB conducted a cross-sectional study on 852 participants from the cohort study based on the Mashhad stroke and heart atherosclerotic disorders (MASHAD study). This study showed a significant correlation between serum anti-HSP27 antibody titers and PAB as well as RDW using Spearman correlation analysis. In addition, the univariate and multivariate logistic regression analyses after adjustment for confounder factors including sex, age, physical activity, and smoking status, showed that the level of anti-hsp27 increased 1.83 fold in line with increasing of 1 unit of PAB in subjects with level of PAB 36.31–82.63 (H2O2%) in comparison to the reference group (PAB level 36.31gt). Our model’s results have shown that PAB was strongly related to serum anti-HSP27 antibody titers.

Our results showed that age was an important and related variables with serum anti-HSP27 antibody titers. Our data showed that serum anti-HSP27 antibody titer did not relate to gender which is consistent with the results of Zilaee et al., and also Rea et al. study^36,37^. Zilaee et al. study was conducted on a total of 106 subjects with metabolic syndrome aged 18–65 years, with and without diabetes based on a case–control study. Rea et al. study was conducted on four age groups (less than 40, between 40 and 69, between 70 and 89, and 90 or larger than 90). No significant differences were observed in anti-HSP antibodies based on gender and age changes. But the results of the regression analysis revealed a significant relation between anti-HSP antibody levels and age^36,37^. In addition, the results of the Kargari et al.^1^ study had similar results to Zilaee et al. and Rea et al. studies about the relationship between age and gender with serum anti-HSP27 antibody titers.

In addition, we observed a strong association with an indirect effect between serum anti-HSP27 antibody titer and the PAL, while Sadabadi et al. found the PAL was not significant. One reason for the discrepancy could be that they performed their study on participants with MetS disease.

Kargari et al. in their study on 933 subjects showed a significant relationship between diabetes mellitus and serum anti-HSP27 antibody titer^1^ that their results were not similar to the results of our research. Azarpazhooh et al.^8^, and Tavana et al.^20^ in their studies reported that diabetes mellitus is not associated with serum anti-HSP27 antibody titer. Azarpazhooh et al.’s study was carried out on 168 patients in the first 24 h after the onset of stroke in a case–control study and the patients were matched in terms of age and gender. Hence, our findings were consistent with their findings. The study of Tavana et al. was conducted based on a case–control study on 106 subjects with metabolic syndrome and 6447 subjects with diabetes mellitus. These differences may be due to the target population, other patient conditions, and time periods for each study.

A study^38^ showed the relationship between BMI and antibody titers to HSP60, 65, and 70 is significant. Kargari et al. found a significant relationship between BMI and HTN with serum anti-HSP27 antibody titer. Also, Azarpazhooh et al. concluded that serum anti-HSP27 antibody titer was significantly higher in hypertensive patients compared with non-hypertensive patients (*p* < 0.001). But, our results showed that BMI and HTN were not associated with serum anti-HSP27 antibody titer. In both studies Kargari et al. and Azarpazhooh et al., no significant difference was observed between smoking status and serum anti-HSP27 antibody titer and was consistent to our conclusion.

Also, we explored the educational level of individuals that was not related to serum anti-HSP27 antibody titer and our result was different from the study of Victora et al.^39^.

In addition to the items mentioned so far, obesity, height, LDL, TG, Chol, WHR, and Hs-CRP were positively associated with serum anti-HSP27 antibody titer in Kargari et al. study. In another study that was conducted by Sadabadi et al., PAL and HC were not significant, and the serum HSP27 antibody titers were different (*p*-value = 0.05) between the subjects with high WC, HDL, TG, BPS, and BPD compared to participants with low WC, HDL, BPS, BPD, and glucose^5^. In our study, PAL was related and consistent with the study of Sadabadi et al.

In addition to all of the above, our finding revealed that there was a relationship between other variables such as MAUC and PLT with serum anti-HSP27 antibody titer. These variables were not studied in previous studies.

In summary, there are differences in the results of this study with other studies mentioned. The previous studies have been conducted using common statistical methods that require special assumptions or case–control studies but in the present study, a non-parametric method that does not require special assumptions has been used as well as it can predict and model the linear and nonlinear relationships between input and output patterns, well. These differences could be due to the cross-sectional study design and sample size in case and control groups^20^. Another reason that can cause these discrepancies is the sample size, which can be influential in the bivariate analysis stage for feature selection in our selection and other studies. Also, the presence of other influential factors or conditions of the subjects, the special patients under study that have been considered in previous studies can be effective. The limitation of this study was the exclusion of important variables such as drug use and vitamin D due to missing values exceeding 70 percent.

## Conclusion

The LightGBM method was effective in elucidating the relationship between PAB and PAL and serum anti-HSP27 antibody titers with a direct and indirect effect on the prediction of serum anti-HSP27 antibody titers, respectively. The PDW, MUAC, SBP, age, RDW, WHR, NL, PLT, glucose, cholesterol, and RBC were also associated with anti-HSP27 antibody titers. In addition, we aim to investigate this topic as a longitudinal study in the future.

## Data Availability

The data that support the findings of this study are available from [Mashhad University of Medical Sciences], but restrictions apply to the availability of these data, which were used under license for the current study, and so are not publicly available. Data are however available from the authors upon reasonable request and with permission of [Mashhad University of Medical Sciences].
